# Genetic and clinical profiles of 160 papillary thyroid cancers with lateral neck lymph node metastasis

**DOI:** 10.3389/fonc.2022.1057532

**Published:** 2023-01-12

**Authors:** Yiqiao Fang, Xun Zheng, Xiuhe Zou, Zi Ye, Jiaye Liu, Jianyong Lei, Zhihui Li

**Affiliations:** ^1^ Department of Thyroid and Parathyroid Surgery, West China Hospital, Sichuan University, Chengdu, Sichuan, China; ^2^ Laboratory of Thyroid and Parathyroid Diseases, Frontiers Science Center for Disease-Related Molecular Network, West China Hospital, Sichuan University, Chengdu, Sichuan, China; ^3^ West China School of Medicine, West China Hospital, Sichuan University, Chengdu, Sichuan, China; ^4^ State Key Laboratory of Biotherapy and Cancer Center, West China Hospital, Sichuan University and Collaborative Innovation Center, Chengdu, Sichuan, China; ^5^ Respiratory Health Institute, Frontiers Science Center for Disease Molecular Network, West China Hospital, Sichuan University, Chengdu, Sichuan, China

**Keywords:** papillary thyroid carcinoma, genetic alternation, lateral lymph node metastasis, BRAF, RET

## Abstract

**Background:**

Lymph node metastasis is widespread in papillary thyroid cancer (PTC). Patients are more vulnerable than those with central lymph node metastasis if they have lateral neck lymph node metastasis (LLNM). There are few researches focus on the correlation between clinical characteristics and genetic profile of PTC with LLNM. In this study, we aimed to analyze the clinical and genetic features of PTC with LLNM.

**Methods:**

A total of 160 primary tumor samples derived from PTC patients with LLNM were involved. Targeted next-generation sequencing was carried out on all samples with 57 known thyroid-cancer-related genes. The associations between genomic alternations and clinical characteristics of PTC with LLNM were statistically evaluated.

**Results:**

The median age of patients was 37 years, ranging from 5 to 77 years and the female/male ratio was 1.86. The most frequently altered genes in our series were *BRAF* mutation (68%), followed by *RET* fusion (17%), *TERT* promoter mutation (5%) and PIK3CA mutation (2%). To be noted, all PTC patients with LLNM of *TERT* promoter mutations appeared along with *BRAF* mutations (8/8) and half of them experienced a relapse. Intriguingly, we found more metastatic lymph nodes in patients with *RET* fusion, but there was no statistically significant difference in metastatic lymph node ratio than those with *BRAF* mutation or without mutation. A high rate of gene fusion (70%) was found in the pediatric population, with aggressive late-onset disease.

**Conclusions:**

PTC patients with LLNM is characterized by a high rate of BRAF mutation. Due to the observed clinicopathological differences in those patients among different alterations, further prospective studies are needed to verify our results and to evaluate the most suitable treatment strategies.

## Introduction

The incidence rate of thyroid cancer has steadily climbed in recent years due to the rapid development of ultrasound imaging (1), making thyroid cancer one of the most prevalent endocrine tumors (2). Papillary thyroid carcinoma (PTC), so named because of its histological architecture, accounts for more than 80 percent of thyroid cancers. PTC typically has a five-year survival of over 95%, making it a docile disease (3). However, there are approximately 30-80% of patients undergoing thyroidectomy for papillary thyroid cancers with clinically meaningful lateral neck lymph node metastases (LLNM). Additionally, studies demonstrate that these patients are more vulnerable (4). Therefore, it is crucial for future treatments to comprehend and depict the clinical characteristics and genetic features (5).

Although newly diagnosed PTCs have risen dramatically, the mortality rate has stayed constant which prompted us to explore which gene alteration may closely correlated with disease progression and patients survival (6). Thyroid cancer is brought on by point mutations or gene rearrangements/fusions of critical genes in thyroid cells that activate the MAPK pathway and PI3K/AKT pathway (7). Therefore, with the in-depth research on the molecular genetics of thyroid cancer, genetic detection has begun to assist the clinical treatment of patients (8). Genetic detection may increase the precision of distinguishing benign from malignant thyroid nodules, forecast cancer risk stratification, and choose targeted drug therapy for advanced thyroid cancer. A comprehensive characterization of the genomic landscape of PTC has been reported by The Cancer Genome Atlas (TCGA) as a homogeneous cohort (9). It demonstrated that PTC had a low frequency of somatic alternations. BRAF (60%) gene mutation is the most common and specific genetic alteration, determining papillary carcinoma’s clinical and pathological manifestations (10). For 20-30% of thyroid nodules that cannot be definitively diagnosed by fine needle aspiration (FNA), thyroid cancer is highly suspected if a BRAF gene mutation is detected. This study dramatically improved our understanding of genotype-phenotype relationships in thyroid cancer. Miguel et al. (11) evaluated the frequency of TERT promoter, BRAF, and NRAS mutations in the primary tumor, lymph node metastases, and distant metastases which showed a high concordance in primary tumor and metastatic sites. However, limited data exist regarding the genetic profile in PTC with LLNM. Dilmi et al. (12) reported the most extensive series of gene-targeted sequencing of PTC with LLNM to date, which focused on papillary microcarcinomas. However, there are few studies on the correlation between clinical characteristics and the genetic profile of PTC with LLNM so far.

Aiming to understand the clinical-molecular correlation and the prognostic factors of PTC with LLNM, we present a sizeable comprehensive retrospective cohort of 160 PTC patients with LLNM underwent surgical resection and targeted NGS at the molecular level in order to provide guidance for clinical treatment and policy makers.

## Materials and methods

### Patients and tissue samples collection

Tumor samples were collected from 160 PTC patients with LLNM between 2019 and 2022 at West China Hospital, Chengdu, China. We obtained informed consent from each patient. West China Hospital’s institutional review board approval was obtained for retrospective data collection and subsequent analyses.

### DNA extraction and targeted NGS

All the samples in this study were fresh tissue samples without any process. We performed simultaneous extraction of tissue DNA using the QIAamp Genomic DNA kit (Qiagen) for downstream genomic analysis according to the manufacturer’s instructions.

Targeted NGS was performed for 57 known thyroid-cancer-related genes shown in **Supplementary Table 1**. These 57 genes were considered to be used for auxiliary diagnosis, prognostic risk assessment, targeted drug assessment, and genetic screening, involving single-nucleotide variation, small fragment insertion or deletion, gene copy number variation, and gene fusion. We used a HiSeqTM 2500 platform (Illumina) for sequencing.

### Definition of metastatic lymph node ratio

Conventionally, the lymph node ratio (LNR) was calculated as the number of metastatic lymph nodes or lymph node metastases (LNM) divided by the number of retrieved lymph nodes or lymph node yield (LNY) (13).

### Statistical analysis

Statistical analyses were performed using R Programming, Graphpad Prism 9 (GraphPad Software, California, CA), and SPSS software 27.0 (IBM Corporation, New York, NY). Categorical relationships were detected using the Chi-square test or Fisher’s exact test. The difference in continuous and discrete variables with normal distribution between groups was analyzed by student’s t-test or two-way ANOVA test. Risk factors of the number of metastatic lymph nodes were calculated from binary logistic regression for univariate and multivariate analysis. A two-sided P. value<0.05 was considered statistically significant.

## Results

### Clinical characteristics of patients and tumor samples

This study collected one hundred and sixty samples from PTC patients with LLNM. **Table 1** summarizes the clinical characteristics of those patients. The age was 37 ± 13.75 years (Mean ± s.d.), ranging from 5 to 77 years. There was a 1.86 female to male ratio (104/56), which indicates a modest female predominance. The tumor (T) stage of patients was mainly T4a (25.00%), followed by T1b (21.25%), T3 (18.75%), T1a (14.38%), T4b (13.75%), and T2 (6.87%). Four patients had distant metastases, and the lung has been the site of all metastatic growths. Eleven patients with lateral neck lymph node metastases presented with disease recurrence. An abnormal index of human thyroglobulin (HTG) was found in 36.25% of the patients due to dysfunction of PTC cells. 25-Hydroxy-Vitamin D (25-OH-VD) in 55.00% of patients was lower than the bottom line of the normal range for several reasons, such as region, sun exposure, dietary habits (**Supplementary Table 2**). The mean maximum dimension of the primary tumor was 19.67mm (3 to 59 mm), with 48.75% demonstrating multifocality lesion within the thyroid gland. Invasion of the thyroid capsule was the most common type of extrathyroidal invasion (88.75%), followed by the invasion to laryngeal nerve (32.50%), neck blood vessels (13.13%), esophagus (6.88%) and trachea (6.88%). In this study, patients had an average of 42 resected lymph nodes (1 to 109 lymph nodes), and 11 metastatic lymph nodes (1 to 41 lymph nodes). 92.50% of patients (148 in 160) also had lymph node metastases in the central region. The mean LNR was 0.29 (0.03-1).

**Table 1:** Clinical characteristics.

Characteristics	Patients (n = 160)
**Age (years)**	36.96 (14)
**Gender (female)**	104 (65%)
T stage	
** 1a**	23 (14%)
** 1b**	34 (21%)
** 2**	11 (7%)
** 3**	30 (19%)
** 4a**	40 (25%)
** 4b**	22 (14%)
**Distant metastasis**	4 (2.5%)
**Maximum dimension of the primary tumor (mm)**	19.67 (12)
Location	
** Left**	40 (25%)
** Right**	57 (36%)
** Both**	63 (39%)
**Multifocality**	78 (49%)
Extrathyroidal invasion	
** Thyroid capsule**	142 (89%)
** Trachea**	11 (7%)
** Esophagus**	11 (7%)
** Recurrent laryngeal nerve**	52 (33%)
** Neck blood vessels**	21 (13%)
Number of metastatic lymph nodes	
** Total**	11 (8)
** Lateral**	6 (5)
** Central**	5 (4)
**Metastatic Lymph Node Ratio**	0.29 (0.03-1)

### Genomic alterations from DNA profiling

**Figure 1:**
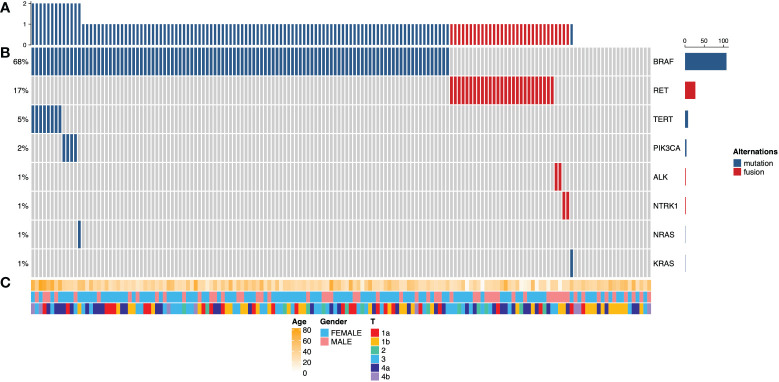
Cancer genome alterations in 160 papillary thyroid carcinomas with lateral lymph node metastasis. **(A)** Mutation density across the PTC with lateral lymph node metastasis cohorts (n = 160), expressed as a number of genetic alterations found in 57 known thyroid-cancer-related genes. **(B)** Oncoprints of PTC with LLNM. The left side shows the percentage of tumors altered for each event; the Color key for genetic alterations is shown in the right panel. **(C)** Clinicopathological features, including patients’ age, gender, and tumor stage. Color keys are shown in the bottom panel.

Among the patients enrolled in this study, targeted NGS of 160 primary thyroid carcinomas detected a median of one (0 to 3) mutation. The frequency of mutations across all cases is shown in **Figure 1**. The most common alternations in our series of PTC with LLNM were *BRAF* mutation (68%), followed by *RET* fusion (17%), which both are genes encoding effectors in the mitogen-activated protein kinase (MAPK) signaling pathway (14). *BRAF* gene mutation plays a vital role in developing PTC, the most common being the V600E subtype (15). Interestingly, none of the patients had co-alterations of *BRAF* and *RET*. Mutations in the promoter of *TERT*, which always co-occurred with *BRAF*, known to upregulate telomerase and promote tumor progression, were detected in 5% of tumors. 13% of patients had not detected any gene alternation in this panel. Mutations of *PIK3CA* in members of the phosphoinositide 3-kinase (PI3K) pathway had also been detected at a low frequency (2%).

### Differences in clinical characters among *BRAF*, *RET*, and non-mutation

**Figure 2:**
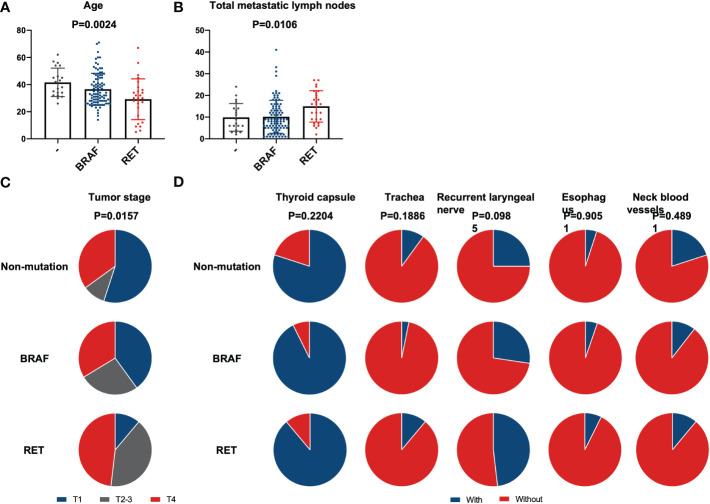
Comparison between *BRAF*, *RET*, and non-mutation. Differences in clinical characteristics between patients with *BRAF* mutation, *RET* fusion, and without mutation. **(A)** Patients’ age; **(B)** Number of lymph node metastases; **(C)** Tumor stage; **(D)** Extrathyroidal invasion. No statistical differences in any extrathyroidal invasion exist between these three groups.

A clinical comparison of patients with alternation of *BRAF* (n=95), *RET* fusion (n=27), and no mutation (n=20) is summarized in **Supplementary Table 3**. The difference in age of groups is statistically significant (ANOVA test, p<0.01). Patients with *RET* fusion were much younger than the other two groups (**Figure 2A**). The group with RET fusion had a slightly increased average number of LNM (15, 2 to 27 lymph nodes) when compared to groups with *BRAF* mutation (10, 1 to 41 lymph nodes) or without gene alternation (10, 3 to 24 lymph nodes) (**Figure 2B**). However, there was no statistical difference in the LNR of the RET group compared to the other 2 groups after the PSM matching (t-test, p=0.40 and 0.98). The proportion of T4 was higher in the *RET* group than other two groups (Chi-square test, p=0.02) (**Figure 2C**). There is no significant difference in the extrathyroidal invasion of the three groups (**Figure 2D**).

### Clinical characters of patients with *TERT* promoter mutation

We identified 8 *TERT* promoter mutations from informative primary tumors. Interestingly, all *TERT* promoter mutations were accompanied by *BRAF* mutations. The age of these patients was 61 ± 12.45 (Mean ± s.d.) and majority of them (7/8) with an advanced tumor stage of T4. Additionally, the largest tumor size in 5 individuals exceeded 40mm, compared to 19.70mm in the entire PTCs with LLNM group. Thus, we supposed that *TERT* promoter mutation aided in the growth of tumor. Besides, four patients with relapse in the TERT promoter mutation group showed a high recurrence rate. *TERT* promoter mutations were rare in PTCs but high-risk and highly prevalent in advanced cancers (16).

### Characters of the pediatric population

This study collected ten samples of primary thyroid tumors from pediatric PTC patients (age < 18 years) with LLNM. The mean age was 10 years, ranging from 5 to 17 years. The most common alternation in pediatric PTC with LLNM was RET fusion (70%), an inherited genetic mutation. The primary tumor stage of patients was mainly T4 (50%), and distant metastases were found in two patients. The mean maximum dimension of the primary tumor was 27.30mm (range, 13 to 45 mm). Invasion of the thyroid capsule was the most common type of extrathyroidal invasion (100%), found in all pediatric patients. Five patients’ tumors had invaded the recurrent laryngeal nerve. Pediatric patients demonstrated a mean of 5 lateral neck metastatic lymph nodes (range, 3 to 16 lymph nodes).

## Discussion

Currently, next-generation sequencing technology simultaneously analyzes hundreds of genes of interest using targeted sequencing panels (17). Understanding the molecular mechanisms of tumor formation is mandatory for accurate diagnoses and personalized treatments (18). Thus, NGS-based molecular tests for oncology research and clinical practice appear rapidly evolving. In PTC, NGS using multigene panels is discussed as an adjunct to ultrasound-guided fine-needle aspiration biopsy to better triage the risk of malignancy in cytologically indeterminate thyroid nodules, potentially reducing the risk of the need for diagnostic surgery with its attendant morbidity (19, 20). It may also contribute to the development of systemic molecular therapies for papillary thyroid cancers that are refractory to standard treatments (21). The discovery of specific genetic alterations and mechanisms of thyroid cancer development is expected to lead to more personalized therapies for patients and achieve precision medicine (22). In addition, the linkage between genetic alterations and clinical features of tumors can be of greater clinical value.

This study evaluated papillary thyroid carcinoma’s mutational status and clinical data of PTC with LLNM. Our result was in accordance with the previous PTC TCGA study (9) that the PTC population had female prominence at 73%. Applying targeted NGS of 57 thyroid-cancer-related genes demonstrates the expected mutations in *BRAF* and *RET*. In our cohort, *BRAF* mutation is the most prevalent alternation, aligned with many landmark researches (9, 23, 24). Tumors driven by *BRAF* do not respond to the negative feedback from ERK to RAF, resulting in high MAPK-signaling (25). There are 27 patients with *RET* fusion common in this study. However, none of the patients had a co-alternation of *BRAF* and *RET*, suggesting that the *BRAF* mutation appears to be an alternative to *RET* in PTC. BRAF mutation and RET fusion may be two mutually exclusive drivers with distinct signaling consequences, like exclusive status between RAS and BRAF mutation (9, 26).

According to our result, patients with *RET* fusion tend to have more lymph node metastases. This result is consistent with that found in the TCGA database (9), with *RET* fusion patients seeing a marginally higher total number of metastatic lymph nodes than *BRAF* mutant patients. To be noted, this discrepancy was more pronounced when compared to the pediatric population (27). Nevertheless, no statistical difference was found in LNR between mutation types after PSM matching. LNR, a way to mitigate the effects of surgery, is computed by dividing the number of metastatic lymph nodes by the total number of lymph nodes evaluated (13). The discrepancy in LNR and absolute values may be related to the insufficient sample size after matching and the limitation of retrospective studies. Only 18 patients in each of the BRAF versus RET groups and 9 patients in each of the non-mutation against RET groups remained after PSM matching. In order to further investigate this data, a bigger sample size is required.

The genetic duet of *BRAF* and *TERT* promoter is rare in the entire population of PTCs (9, 28, 29). A genetic-clinical correlation study demonstrated PTCs with a disease-specific mortality risk order of the genetic duet>>>>BRAF V600E alone = TERT promoter mutation alone > wild-type for both genes (29). It presents an essential indication in papillary thyroid cancers with clinically aggressive features and advanced disease (30, 31). Particularly, *BRAF* and *TERT* promoter mutational statuses have already been included as a criterion for the risk stratification system in the American Thyroid Association guidelines (32). Our study is consistent with the proposed mechanism, whereby the *TERT* promoter mutations generate *de novo* binding elements for ETS-family transcription factors activated by MAPK signaling (33).

We included 10 pediatric patients in this study. It is well known that pediatric thyroid cancer has potential molecular and biological differences compared to thyroid cancer in adults. Specifically, our study found a high rate of gene fusion in the pediatric population, up to 70% in line with other studies (27, 34), compared to 15% in the adult population. The aggressive late-onset disease is more frequent in children than in adults (35). It often spreads to the lymph nodes in the neck and may also spread to the lung (36). Lung metastases were also found in 2 patients in our study (2/10), which is more common than adult thyroid cancer. However, additional efforts with broader groups and larger cohorts are needed if we are to better define the genomic landscape of pediatric thyroid cancer and whether there is an association between histology and/or outcome.

We conduct a contemporary large-scale clinical and molecular cohort with a clinically meaningful group of PTCs with LLNM and analyze the relationship between clinical data and genetic profile. LLNM is always known as a higher risk. Lee et al. (4) evaluated the effect of lymph node-related factors on the risk and location of recurrence in patients with PTCs with LLNM. There was no analysis of genetic detection talked about in Lee’s study. Zheng et al. (37) analyzed factors including clinical results, pathology records, ultrasound results, and *BRAFV600E* status of PTC and found risk factors for cervical lymph node metastasis. However, it explained the relationship between clinical data and *BRAF* mutation rather than a genetic profile. Thus, the present investigation suffered from limitations inherent to the lack of comprehensive clinical data and polygenic detection results. However, our study spends a lot of time and effort to collect exhaustive patient information and perform targeted NGS on large primary tumor samples from 2019 to 2022. And advanced patients and patients with distant metastasis are included in this study, which is close to the constitution of PTC with lateral neck lymph node metastasis group. Thus, this study cohort comprised a large-size group of patients with several advanced samples and comprehensive clinical and genetic information, which may help to make a more sensible clinical decision.

This study analyzes the most frequently thyroid-cancer-related mutated gene. Further studies will be required to apply highly sensitive sequencing techniques to evaluate more oncogenic mutations and whether these results can be verified in a larger sequencing panel. Furthermore, we do not explore the basic mechanism of the clinical difference shown in different genotypes. Thus, a lot of room for improvement is given to this research.

## Conclusions

In conclusion, we analyzed the clinical and genetic data from a cohort of PTCs with LLNM in a comprehensive manner. We identified the relationship between certain genotype and clinical feature. *RET* fusion is associated with more metastatic lymph nodes but no significant increase on metastatic lymph node ratio. And the genetic duet of *BRAF* and *TERT* was related to advanced cancers. Such an effort may help to make a more sensible clinical decision in the treatment of PTC with LLNM.

## Data availability statement

The raw data supporting the conclusions of this article will be made available by the authors, without undue reservation.

## Author contributions

Concept and design: YF, XZ. Acquisition of data: ZY, XHZ, JLe. Analysis and interpretations of data: YF, ZL. Drafting of the manuscript: YF. Critical revision of the paper for important intellectual content: JLi, XZ, XHZ. Supervision: ZL, JLi. All authors contributed to the article and approved the submitted version.
