# Supplementary material for: Genetic and clinical profiles of 160 papillary thyroid cancers with lateral neck lymph node metastasis
Source: Front Oncol. 2023 Jan 12;12:1057532. doi: 10.3389/fonc.2022.1057532 (PMC9877400; doi:10.3389/fonc.2022.1057532)
Supplement: Supplementary file 1 [file DataSheet_1.zip › Supplementary materials/FangSupplementaryTable2.docx]

**Supplementary Table 2.** Clinical characteristics of blood tests before surgery

| **Characteristics** | **Patients(n=160)** |
| --- | --- |
| **FT3** (abnormal rate) | 4.86(3.1%) |
| **FT4** (abnormal rate) | 16.12(7.5%) |
| **TSH** (abnormal rate) | 3.13(21.3%) |
| **CEA** (abnormal rate) | 1.66(1.3%) |
| **PTH** (abnormal rate) | 5.35(18.8%) |
| **Tg** (abnormal rate) | 148.7 (36.3%) |
| **25-OH-VD** (abnormal rate) | 47.21(55%) |
| **Ca** (abnormal rate) | 2.32(5%) |
