# Supplementary material for: Genetic and clinical profiles of 160 papillary thyroid cancers with lateral neck lymph node metastasis
Source: Front Oncol. 2023 Jan 12;12:1057532. doi: 10.3389/fonc.2022.1057532 (PMC9877400; doi:10.3389/fonc.2022.1057532)
Supplement: Supplementary file 1 [file DataSheet_1.zip › Supplementary materials/FangSupplementaryTable3.docx]

**Supplementary Table 3**. Comparison for clinical characteristics between *BRAF*, *RET*, and Non-mutation

| **Characteristics** | ***BRAF***  **(n=95)** | ***RET***  **(n=27)** | **Non-mutation**  **(n=20)** | **P** |
| --- | --- | --- | --- | --- |
| **Age (years)** | 36.54(11.76) | 29.22(15.03) | 41.65(10.46) | **0.0024** |
| **Gender (female)** | 68(74.7%) | 16(59.3%) | 11(55%) | 0.2311 |
| **T stage** | | | | |
| **1** | 38(40%) | 3(11%) | 11(55%) | **0.0157** |
| **2-3** | 25(26%) | 11(41%) | 2(10%) |  |
| **4** | 32(34%) | 13(48%) | 7(35%) |  |
| **FT3** | 4.79(0.62) | 5.22(1.26) | 4.73(0.62) | **0.0304** |
| **FT4** | 15.75(2.27) | 16.67(3.41) | 16.08(1.67) | 0.2281 |
| **TSH** | 3.17(2.07) | 3.16(2.04) | 3.09(1.37) | 0.9861 |
| **Normal** | 73(76.8%) | 21(77.8%) | 17(85%) | 0.7235 |
| **Abnormal** | 22(23.2%) | 6(22.2%) | 3(15%) |  |
| **CEA** | 1.63(1.08) | 1.56(1.08) | 1.75(1.02) | 0.8427 |
| **PTH** | 5.38(1.41) | 4.74(2.24) | 5.38(1.41) | 0.2675 |
| **Normal** | 77(81.1%) | 22(81.5%) | 17(85%) | 0.9171 |
| **Abnormal** | 18(18.9%) | 5(18.5%) | 3(15%) |  |
| **Tg** | 80.11(157.2) | 299.3(993.1) | 31.91(37.85) | 0.0607 |
| **<1.4** | 12(12.6%) | 5(20%) | 2(10%) | 0.1363 |
| **>1/4, <78** | 63(66.3%) | 11(44%) | 16(80%) |  |
| **>78** | 20(21.1%) | 9(36%) | 2(10%) |  |
| **25-OH-VD** | 45.82(16.26) | 49.05(19.62) | 50.13(20.88) | 0.5030 |
| **Normal** | 37(39.8%) | 11(45.8%) | 9(45%) | 0.8194 |
| **Abnormal** | 56(60.2%) | 13(54.2%) | 11(55%) |  |
| **Ca** | 2.32(0.09) | 2.30(0.35) | 2.31(0.07) | 0.8074 |
| **Maximum dimensions of the primary tumor (mm)** | 17.88(10.99) | 22.38(11.76) | 18.25(9.74) | 0.1784 |
| **Location** | | | | |
| **Left** | 25(26.3%) | 9(33.3%) | 2(10%) | 0.1348 |
| **Right** | 28(29.5%) | 10(37.0%) | 11(55%) |  |
| **Both** | 42(44.2%) | 8(29.6%) | 7(35%) |  |
| **Multifocality** | 49(51.6%) | 13(48.1%) | 7(35%) | 0.4024 |
| **Extrathyroidal invasion** |  |  |  |  |
| **Thyroid capsule** | 88(92.6%) | 24(88.9%) | 16(80%) | 0.2204 |
| **Trachea** | 3(3.2%) | 3(11.1%) | 2(10%) | 0.1886 |
| **Esophagus** | 5(5.3%) | 2(7.4%) | 1(5%) | 0.9051 |
| **Recurrent laryngeal nerve** | 26(27.4%) | 13(48.1%) | 5(25%) | 0.0985 |
| **Neck blood vessels** | 10(10.5%) | 3(11.1%) | 4(20%) | 0.4891 |
| **Number of metastatic lymph nodes** | | | | |
| **Total** | 10.17(7.62) | 14.96(7.24) | 9.9(6.39) | **0.0106** |
| **Lateral** | 5.84(4.96) | 7.22(4.68) | 5.6(4.70) | 0.3887 |
| **Central** | 4.33(3.84) | 7.74(4.86) | 4.3(3.9) | **0.0006** |
