# Supplementary material for: Genetic and clinical profiles of 160 papillary thyroid cancers with lateral neck lymph node metastasis
Source: Front Oncol. 2023 Jan 12;12:1057532. doi: 10.3389/fonc.2022.1057532 (PMC9877400; doi:10.3389/fonc.2022.1057532)
Supplement: Supplementary file 1 [file DataSheet_1.zip › Supplementary materials/FangSupplementaryTable1.docx]

**Supplementary Table 1**. List of 57 target genes

| AKT1 | AKT2 | ALK^*^ | APC | ATM |
| --- | --- | --- | --- | --- |
| AXIN1 | BRAF | CCDC6^*^ | CDKN1B | CDKN2A |
| CDKN2B | CDKN2C | CHEK2 | CTNNB1 | EGFR |
| EIF1AX | EML4^*^ | ERBB2 | EZH1 | FGFR2^*^ |
| GNAS | HRAS | IDH1 | KIT | KRAS |
| MED12 | MEN1 | MET | NCOA4^*^ | NCOR2 |
| NF1 | NF2 | NOTCH1 | NRAS | NTRK1^*^ |
| OFD1^*^ | PAX8^*^ | PDGFRA | PDGFRB | PIK3CA |
| PPARγ^*^ | PRKAR1A^*^ | PTEN | RAC1 | RB1 |
| RBM10 | RET^*^ | STOP | STK11 | STRN^*^ |
| TERT | TFG^*^ | TG | TP53 | TPM3^*^ |
| TSHR | ZNF148 |  |  |  |

* gene fusion
